# Prognostic significance of DNA repair proteins MLH1, MSH2 and MGMT expression in non-small-cell lung cancer and precursor lesions

**DOI:** 10.1111/j.1365-2559.2008.02999.x

**Published:** 2008-04-01

**Authors:** W A Cooper, M R J Kohonen-Corish, C Chan, S Y Kwun, B McCaughan, C Kennedy, R L Sutherland, C-S Lee

**Affiliations:** 1 Department of Anatomical Pathology, Royal Prince Alfred Hospital Sydney, Australia; 2 Cancer Research Programme, Garvan Institute of Medical Research and St Vincent's Clinical School, Faculty of Medicine, University of NSW Sydney, Australia; 3 Department of Anatomical Pathology, Concord Hospital Sydney, Australia; 4 Department of Cardiothoracic Surgery, Royal Prince Alfred Hospital Sydney, Australia; 5 Strathfield Private Hospital Sydney, Australia; 6 Cancer Pathology Laboratory, Bosch Institute and Discipline of Pathology, University of Sydney Sydney, Australia

**Keywords:** DNA mismatch repair proteins, immunohistochemistry, MGMT, MLH1, MSH2, non-small-cell lung cancer, prognosis

## Abstract

**Aims:**

To investigate the role of DNA repair proteins and their prognostic significance in non-small-cell lung cancer (NSCLC).

**Methods and results:**

A retrospective analysis of 108 cases of stage I–II NSCLC was undertaken. Immunohistochemical expression of DNA repair proteins MLH1, MSH2 and MGMT was assessed using tissue microarrays of paraffin-embedded samples of invasive carcinoma and precursor lesions. Results were analysed in relation to clinicopathological parameters and patient survival. Reduced expression of MLH1 was found in 58.5% of tumours and occurred less frequently in poorly differentiated tumours (*P* = 0.044) and large cell carcinomas (*P* = 0.004). MSH2 and MGMT expression was reduced in 18.1% and 77.8% of cases, respectively. There was an inverse relationship between MLH1 and MSH2 expression (*P* = 0.012). Normal expression of MLH1, MSH2 and MGMT was found in all cases of squamous metaplasia and squamous dysplasia. Only a single case of carcinoma *in situ* (12.5%) showed reduced MLH1, none showed reduced MSH2 and 25% showed reduced MGMT. Survival analyses showed no prognostic significance based on expression of MLH1 (*P* = 0.92), MSH2 (*P* = 0.78) or MGMT (*P* = 0.57).

**Conclusions:**

Reduction in expression of DNA repair proteins MLH1, MSH2 and MGMT is relatively common in NSCLC, appears to be a late event in the development of invasive malignancy and does not influence survival in this patient cohort.

Cooper W A, Kohonen-Corish M R J, Chan C, Kwun S Y, McCaughan B, Kennedy C, Sutherland R L & Lee C-S (2008) *Histopathology***52,** 613–622

Prognostic significance of DNA repair proteins MLH1, MSH2 and MGMT expression in non-small-cell lung cancer and precursor lesions

## Introduction

The role of DNA mismatch repair (MMR) proteins in sporadic and hereditary colorectal carcinoma has been extensively investigated,^1^ but the role of these proteins in the molecular pathogenesis of non-small-cell lung cancer (NSCLC) is poorly understood. Alterations in DNA produced during replication and recombination are repaired by the MMR system in an effort to maintain genomic stability, and tumours lacking MMR function exhibit a mutator phenotype.^2^ In addition, the cytotoxic effects of a number of alkylating agents used in the treatment of cancer are dependent on a functional MMR system.^2^ Hereditary non-polyposis colorectal cancer (HNPCC/Lynch syndrome) is an autosomal dominant condition, which comprises 2–5% of all colorectal cancers. It results from germ-line mutations in *MMR* genes, with alterations of hMLH1 and hMSH2 accounting for the vast majority of cases.^1^ This syndrome is characterized by genetic instability and the propensity to develop a number of neoplasms, particularly colorectal cancer and, to a lesser extent, malignancies of the endometrium, stomach, pancreas, ureters, ovaries, brain and skin.^3^ Pulmonary neoplasms are not a characteristic feature of this syndrome, suggesting that defective *MMR* gene function may not play a major role in the pathogenesis of NSCLC. Interestingly, alterations in expression of MMR proteins MLH1 and MSH2 have been reported in a variable proportion of NSCLC ranging from 18%^4^ to 61%,^5^ but no studies have investigated the role of reduced protein expression in precursor lesions of NSCLC and very few have investigated their potential prognostic significance in invasive carcinomas.

Methyl guanine DNA methyltransferase (MGMT) is a DNA repair enzyme involved in removal of abnormal adducts from the O^6^ position of guanine, providing protection from mutagenic agents and conferring resistance to alkylating chemotherapeutic drugs.^2^ MGMT expression is thought to be induced by a number of toxic agents, including cigarette smoke.^6^ Promoter region methylation resulting in reduced expression of MGMT occurs commonly in a variety of tumours such as colorectal cancer and melanoma^7^,^8^ and has been reported as an unfavourable prognostic factor in NSCLC.^9^ However, very few studies have investigated the role of altered MGMT protein expression in NSCLC.

In this study, expression of DNA repair proteins MLH1, MSH2 and MGMT were investigated in early-stage NSCLC and precursor lesions using tissue microarrays (TMAs) and the results have been correlated with clinicopathological parameters and patient survival.

## Materials and methods

### Patient cohort

Tumour samples and clinical follow-up data were obtained from a cohort of 108 stage I–II NSCLC patients treated at the Royal Prince Alfred Hospital, Sydney, Australia between 1997 and 1999. The cohort included 70 (64.8%) men and 38 women (35.2%) with a median age at diagnosis of 67 years (range 41–81 years) and median survival time of 72 months (range 3.3–97.5 months), excluding patients with survival <60 days. Histological tumour subtypes were assessed using the World Health Organization classification,^10^ and there were 48 (44.4%) adenocarcinomas (ADCs) [including seven bronchioloalveolar carcinomas (BACs)], 19 (17.6%) large cell carcinomas (LCCs), 40 (37.0%) squamous cell carcinomas (SCCs) and one (0.9%) mixed adenosquamous carcinoma. For survival analyses, invasive ADCs and (non-invasive) BACs were assessed both separately and as a single group. Tumours were staged using the American Joint Committee on Cancer Tumor-Node-Metastasis classification^11^ and consisted of 88 (81.5%) stage Ι and 20 (18.5%) stage ΙΙ tumours. Regional lymph node metastases were available from nine patients. Precursor lesions were also assessed when available, and there were up to 13 cases of bronchial squamous epithelial metaplasia, two with low-grade dysplasia, and eight cases of bronchial SCC *in situ*; however, there was insufficient material for adequate assessment of all of these cases with all three markers. Follow-up information of at least 5 years was available for this study.

### Tumour samples

TMAs were constructed using three to four donor cores of tumour, 1 mm in diameter, from appropriate areas in formalin-fixed paraffin-embedded tissue blocks. These tissue cores were arrayed into a recipient paraffin block using a tissue arraying instrument (Beecher Instruments, Silver Springs, MD, USA). Serial sections were cut from the TMA blocks at 4 μm thickness and mounted on glass slides. The use of TMAs to investigate immunohistochemical expression of MLH1 and MSH2 as opposed to whole sections of tumours has been validated in a study of colorectal carcinomas.^12^

### Immunohistochemistry

Sections were deparaffinized with xylene and rehydrated through graded alcohols to water. Immunohistochemical analysis for protein expression of the *MGMT*, *MLH1* and *MSH2* genes was undertaken using the following antibodies. MLH1 (clone G168-15; BD Pharmingen, San Diego, CA, USA; diluted 1:100), MSH2 (clone FE11; Oncogene, San Diego, CA, USA; diluted 1:1000) and MGMT (clone MT23.2; Zymed, Carlsbad, CA, USA; diluted 1:600). Immunohistochemistry was performed using Goat Anti-Mouse IgG, Polymer-Horseradish Peroxidase IHC amplification reagent (Chemicon, Temecula, CA, USA) as the detection system and 3,3′-diaminobenzidine as the substrate chromogen (ICN Biomedicals, Aurora, OH, USA). Heat-induced antigen retrieval was performed by heating in a pressure cooker (Decloaking Chamber; Biocare Medical, Concord, CA, USA) in preheated citrate buffer (10 mmol/l, pH 6.0) for 5 min. In some cases, the tissue sections were microwave treated in preheated ethylenediamine tetraaceticacid buffer for 20–40 min. All immunohistochemistry was performed on a Sequenza rack with Coverplate (ThermoShandon, Pittsburgh, PA, USA). The slides were treated with 1% goat serum and then incubated with primary antibody overnight at room temperature. Upon completion of a Tris buffer wash, the slides were incubated with enzyme-conjugated polymer with goat antimouse IgG antibody for 30 min at room temperature and then washed in buffer. After reaction with diaminobenzidene/hydrogen peroxide for 5 min, slides were rinsed in distilled water and immersed in 1% copper sulphate solution for 1 min. After washing, the sections were counterstained in Gill's Haematoxylin 2 (Australian Biostain, Traralgon, Australia) solution for 30 s, followed by blueing solution for 15 s.

The positive controls were matched samples of normal bronchial mucosa and peripheral lung parenchyma incorporated into the tissue arrays. Samples of normal bronchial mucosa showed positive staining for MMR proteins in an average of 95–100% of cells in all cases except for two cases stained with MLH1, which were excluded from the analysis. Samples from normal spleen were also incorporated into the arrays to use as both external positive controls and also as reference points within the arrays. Nuclear expression of MLH1 was also seen in all splenic samples, but the reactivity was not scored. A negative control slide was incubated with non-immune serum instead of the primary antibody.

### Scoring

Two pathologists (W.A.C. and C.C.) independently scored each case without knowledge of the patient's clinical details and an average of the two scores obtained was used. Immunohistochemical expression of MLH1 was scored semiquantitatively by multiplying the percentage of cells showing nuclear expression and the intensity of immunoreactivity using a three-tier grading system (1 = weak, 2 = moderate and 3 = strong). An average score was obtained from the multiple samples of each case and reduced protein expression was taken as cases with a score of <200. Where markedly discrepant, the case was reviewed before deciding on an appropriate score. There was good correlation between the scores obtained from each pathologist (MLH1 correlation coefficient *R* = 0.94, *P* < 0.01; MSH2 *R* = 0.79, *P* < 0.001 and MGMT *R* = 0.96, *P* < 0.001).

### Statistical analyses

The Pearson χ^2^ test and Fisher's exact test (two-sided) were used to compare associations between protein expression and various clinicopathological characteristics. The Kaplan–Meier log rank and Cox proportional regression model were used for survival analyses. SPSS statistical software package version 13.0 was used for all analyses (SPSS Inc., Chicago, IL, USA). *P*-values of <0.05 were regarded as statistically significant.

## Results

### MMR protein expression

**Figure 1:**
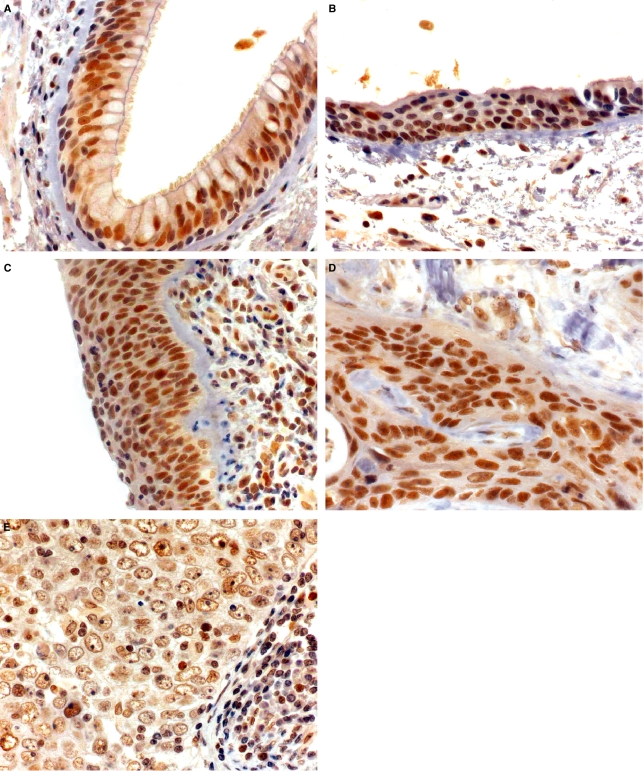
Immunohistochemistry for MLH1. Strong expression of MLH1 is seen in (**A**) normal bronchial epithelium, (**B**) bronchial epithelium with squamous metaplasia, (**C**) bronchial epithelium with carcinoma *in situ* (most cases) and most invasive squamous cell carcinomas (SCCs) (**D**). Some cases of invasive SCC show reduced expression of MLH1 (**E**).

Nuclear expression of MLH1 was seen extensively in normal tissues, but was reduced in 62 out of the 106 cases of NSCLC (58.5%) (Figure 1). In SCC, MLH1 expression was reduced in 27/39 (69.2%), in LCCs 5/18 (27.8%) and in ADCs 30/48 (62.5%), including 5/7 (71.4%) BACs. Of eight cases of bronchial epithelial squamous carcinoma *in situ*, only one case (12.5%) showed reduced MLH1 expression. Thirteen cases of bronchial epithelial squamous metaplasia were assessed and none had reduced expression of MLH1, including two cases with low-grade dysplasia. Of nine cases with lymph node metastases, only two (22.2%) showed reduced MLH1 expression.

**Figure 2:**
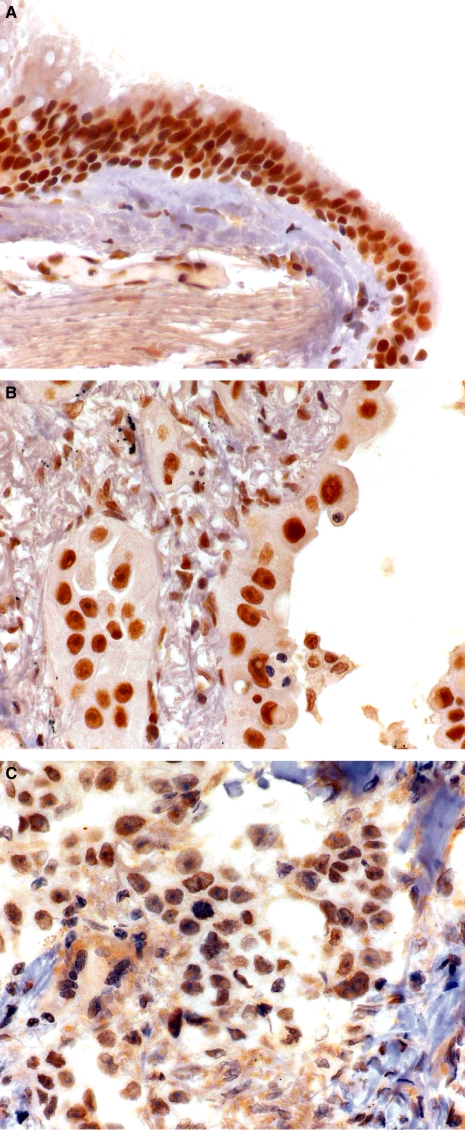
Immunohistochemistry of MSH2 in (**A**) normal bronchial epithelium, (**B**) adenocarcinoma with normal expression of MSH2, and (**C**) adenocarcinoma with reduced expression of MSH2.

There was reduced MSH2 expression in 19 of 105 cases (18.1%) (Figure 2). In different histological subtypes, MSH2 was reduced in 4/39 SCCs (10.3%), 3/16 (18.8%) LCCs and 11 of 48 (22.9%) ADCs, including 2/7 (28.6%) BACs. MSH2 was not reduced in any of the seven cases of bronchial epithelial squamous carcinoma *in situ* or any of the 11 cases of squamous metaplasia with or without dysplasia in bronchial epithelium. There was a total of nine lymph node metastases, with none showing reduced MSH2 expression.

**Figure 3:**
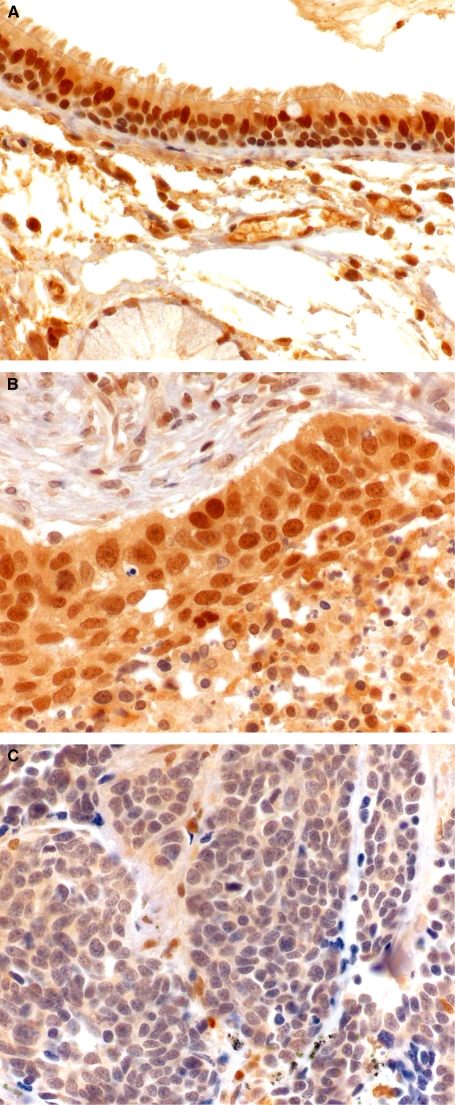
Immunohistochemistry of methyl guanine DNA methyltransferase (MGMT) in (**A**) normal bronchial epithelium, (**B**) large cell carcinoma with normal expression of MGMT, and (**C**) large cell carcinoma with reduced expression of MGMT.

Reduced MGMT scores were seen in 84 of 108 cases (77.8%) of NSCLC (Figure 3). In SCC, 32 (80%) showed reduced expression of MGMT, LCC 13 (68.4%) and in ADCs 38 (79.2%). In eight cases of bronchial epithelial squamous carcinoma *in situ*, there was reduced MGMT expression in two (25%) cases. Samples of bronchial epithelial squamous metaplasia did not show any reduction in MGMT reactivity in the eight cases (0%) without dysplasia or the two cases (0%) with low-grade dysplasia. Of nine lymph node metastases, four cases (44.4%) had reduced expression of MGMT.

### Correlations between expression of different proteins

Reduction of either MLH1 or MSH2 was found in 64/104 cases (61.5%) and co-reduction of both MLH1 and MSH2 proteins was found in 16 cases (15.4%). There was an inverse relationship between expression of the two proteins that was statistically significant (*P* = 0.012). Ninety-two of 106 (86.8%) showed reduction of either MLH1 or MGMT, and 52 cases (49.1%) showed reduction of both MLH1 and MGMT. Eighty-three of 105 (79.0%) showed reduction of either MSH2 and MGMT, and 17 (16.2%) showed reduction of both of these proteins.

### Correlations with pathological and clinical variables

Reduced expression of MLH1 was found to be less common in LCCs (*P* = 0.004, Pearson's χ^2^; *P* = 0.007, Fisher's exact test two-sided) and poorly differentiated tumours (*P* = 0.044, Pearson's χ^2^) (Table 1). Other pathological or patient factors such as gender, age, tumour size, stage, lymphatic invasion, blood vessel invasion, perineural invasion and involvement of the bronchial surgical margin were evenly distributed between those with and without reduced MLH1 expression. MSH2 and MGMT expression did not correlate with any of the measured clinicopathological parameters (Table 1).

**Table 1:** Relationship between protein expression and clinicopathological characteristics of patients

	MLH1 expression			MSH2 expression			MGMT expression		
			
	Normal	Reduced	χ^2^, *P*-value	Normal	Reduced	χ^2^, *P*-value	Normal	Reduced	χ^2^, *P*-value
Tumour type			0.028*			0.29			0.64
ADC	16	24	0.81	37	11	0.24	10	37	0.84
BAC	2	5	0.47	5	2	0.46	2	5	0.68
SCC	12	27	0.087	35	4	0.11	8	32	0.67
LCC	13	5	0.004*	13	3	0.94	6	13	0.28
Mixed	1	1	†	1	1	†	0	2	†
Differentiation			0.11			0.84			0.61
Well	2	6	0.32	6	2	0.60	3	6	0.40
Mod	20	37	0.15	50	10	0.66	11	46	0.44
Poor	22	19	0.044*	30	7	0.87	10	32	0.75
Size, mm			0.79			0.39			0.33
≤30	23	34		45	12		15	43	
>30	21	28		41	7		9	41	
Sex			0.17			0.95			0.24
Male	32	37		55	12		18	52	
Female	12	25		31	7		6	32	
Age, years			0.74			0.87			0.61
<67	22	33		39	9		10	40	
≥67	22	29		47	20		14	44	
Stage			0.76			0.84			0.47
1A	11	19	0.52	24	7	0.44	9	22	0.28
1B	23	33	0.92	46	8	0.37	10	47	0.22
2A	3	2	0.39	4	1	0.91	2	3	0.33
2B	7	8	0.66	12	3	0.84	3	12	0.82
BVI			0.81			0.14			0.28
Absent	41	57		81	16		21	79	
Present	3	5		5	3		3	5	
LVI			0.32			0.60			0.84
Absent	42	56		80	17		22	78	
Present	2	6		6	2		2	6	
PNI			0.23			0.24			0.45
Absent	44	60		5	18		24	82	
Present	0	2		1	1		0	2	
Margin			0.39			0.20			0.60
Not involved	40	59		79	19		23	78	
Involved	4	3		7	0		1	6	

* Statistically significant (*P* < 0.05).

† Too few cases to assess.

ADC, Adenocarcinoma; BAC, bronchioloalveolar carcinoma; SCC, squamous cell carcinoma; LCC, large cell carcinoma; BVI, blood vessel invasion; LVI, lymphovascular invasion; PNI, perineural invasion.

### MMR protein expression and patient survival

**Figure 4:**
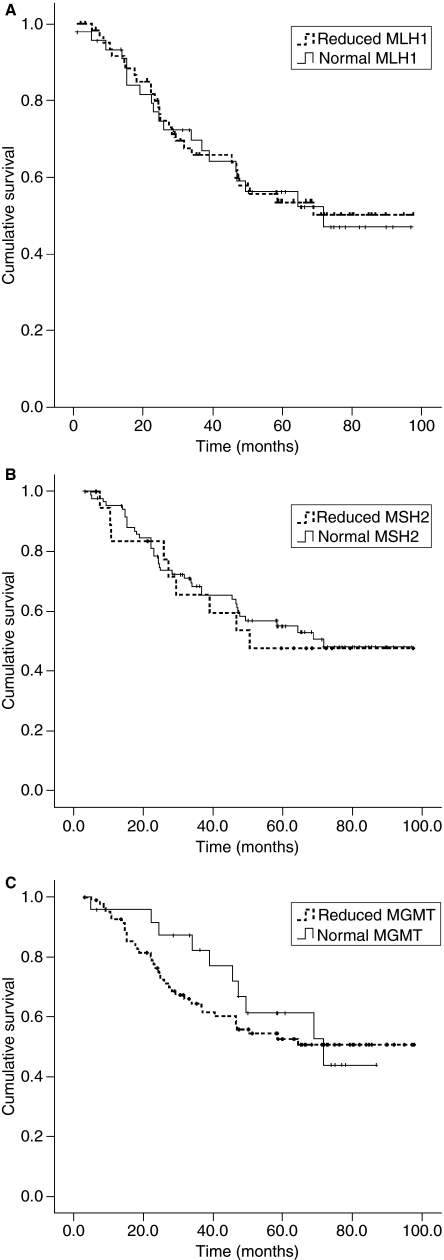
**A**, Probability of survival according to MLH1 expression [Kaplan–Meier survival curve, *P* = 0.92, log rank (Mantel–Cox)]. Reduced MLH1 expression *n* = 62 (dotted line), normal MLH1 expression *n* = 44 (solid line). There were 60 censored cases. **B**, Probability of survival according to MSH2 expression using immunohistochemical score (Kaplan–Meier survival, *P* = 0.78, log rank test). Reduced MSH2 expression *n* = 19 (dotted line), normal MSH21 expression *n* = 86 (solid line). There were 58 censored cases. **C**, Probability of survival according to MGMT expression [(Kaplan–Meier survival curve, *P* = 0.57, log rank (Mantel–Cox); *P* = 0.25, Breslow; *P* = 0.36, Tarone–Ware]. Reduced MGMT expression *n* = 83 (dotted line), normal MGMT expression *n* = 24 (solid line). There were 60 censored cases.

Expression of MMR proteins was compared with overall patient survival using Kaplan–Meier survival analysis. No significant correlation was found between survival and expression of MLH1 (*P* = 0.92), MSH2 (*P* = 0.78) or MGMT (*P* = 0.57) (Figure 4). Tumours that showed a reduction of either MLH1 or MSH2 (or both) were not associated with survival (*P* = 0.83). Similarly, other combinations of DNA repair protein expression (MLH1 and/or MGMT reduced, MSH2 and/or MGMT reduced) did not correlate with survival (data not shown). Analysis of survival based on reduced MMR protein expression using the Kaplan–Meier method was not significant when the data were subanalysed based on tumour grade, histological type, stage or patient gender (data not shown). Similarly, using Cox regression analysis, no significant correlation was found between survival and expression of MLH1 [*P* = 0.94, hazard ratio (HR) 0.98, 95% confidence interval (CI) 0.54, 1.76], MSH2 (*P* = 0.78, HR 0.90, 95% CI 0.44, 1.87) or MGMT (*P* = 0.57, HR 0.82, 95% CI 0.41, 1.6).

## Discussion

In this study, expression of DNA repair proteins MLH1, MSH2 and MGMT have been investigated in early-stage NSCLC and precursor lesions using TMAs. Although the role of MMR proteins in sporadic and hereditary colorectal carcinoma has been extensively investigated,^1^ the role of these proteins in the molecular pathogenesis of lung cancer is poorly understood. Molecular alterations of *MMR* genes have been found in a significant number of NSCLCs, and promoter methylation is thought to be the predominant mechanism of silencing *hMLH1* and *hMSH2* genes in these cases.^4^,^13^,^14^ Some studies have found that loss of heterozygosity at loci for DNA *MMR* genes is relatively frequent in NSCLC,^15^,^16^ whereas others have not been able to demonstrate *hMLH1* promoter methylation.^17^ Homozygous deletions or rearrangements have not been demonstrated in the *hMLH1* gene in NSCLC.^15^,^16^ In this study, we have demonstrated that decreased DNA repair protein expression is relatively common in NSCLC with MLH1, MSH2 and MGMT reduced in 58.5%, 18.1% and 77.8% of NSCLC cases, respectively. Reduced expression of MLH1 in NSCLC has been reported at frequencies of 20–61%,^5^,^18^ and in some studies has been associated with microsatellite instability.^5^,^18^ Decreased MSH2 expression has been reported at similar rates ranging from 18%^4^ to 58%,^16^ and in our study population altered MSH2 was less frequent than MLH1. The differences between studies possibly relate to different patient populations and different criteria being applied to determine reduction of protein expression.

In a study of 150 cases of NSCLC, Xinarianos *et al.*^16^ have found reduced expression of MLH1 protein in 58.6% of cases, MSH2 in 57.8% and either MLH1 or MSH2 in 82%. Although we have found reduced expression of MSH2 in considerably fewer cases, our results for MLH1 and reduction of either protein were similar. They did not find any correlation between reduced MLH1 expression and age, gender, tumour differentiation or TNM T stage, but they did demonstrate an association with heavy smoking and nodal metastases in SCC.^16^ In contrast, in our study reduced MLH1 was less common in poorly differentiated tumours and large cell-type carcinomas. Interestingly, colorectal carcinomas associated with altered MMR protein function are associated with a variety of clinicopathological characteristics, including a propensity to be poorly differentiated.^19^,^20^ As in our study, Xinarianos *et al.*^16^ have not found hMSH2 expression to be correlated with any of the clinicopathological parameters assessed.

Whereas others have reported no loss of expression of MMR proteins MLH1, MSH2 or MSH6 in the non-invasive bronchioloalveolar subtype of ADC,^21^ we were able to demonstrate reduction of MLH1 in 71.4% and MSH2 in 28.6% of this type of carcinoma. Our results for this subset of ADC are very similar to our findings in all ADCs, suggesting the role of altered DNA MMR function in both types of tumour, if any, is likely to be similar. The investigation reported by Aubry *et al.*^21^ did not include invasive ADCs, criteria used for assessing immunohistochemical expression were not clearly defined, and it is possible that methodological differences could account for the discrepant findings.

MGMT promoter region methylation has been demonstrated in 7–55% of NSCLC^14^,^22^,^23^ and is an independent predictor of poor prognosis in one study,^9^ whereas others have not been able to demonstrate a significant association with survival.^24^ Immunohistochemical expression of MGMT has been studied in a group of 83 stage I–III NSCLC and reduced expression was found in only 25% of cases,^6^ compared with 77.8% in our study. There was a significant difference in MGMT expression between smokers and non-smokers (in whom the protein was more frequently reduced)^6^ and between ADC and LCC, with none of the LCCs showing loss of expression, whereas in our study we did not demonstrate any association with tumour type and found loss of expression in a considerable number of LCCs (68.4%). As in our study, they found no association between MGMT expression and age, gender, stage or histological tumour type.

The development of pulmonary SCC is known to occur through a stepwise progression of bronchial epithelial abnormalities starting from squamous metaplasia through to dysplasia, carcinoma *in situ* and, ultimately, invasive carcinoma. Although the histomorphological changes in this process are recognized, the underlying molecular alterations are only poorly understood. Early alterations include overexpression of cyclin D1, cyclin E and p53, whereas loss of retinoblastoma expression occurs late in the development of invasive carcinoma.^25^ We found that although reduced expression of MLH1, MSH2 and MGMT was not uncommon in invasive NSCLC, it was relatively rare in precursor lesions, with no reductions seen in the earliest histological abnormalities of metaplasia and dysplasia. Only very few cases of carcinoma *in situ* showed reduced expression of the DNA repair proteins, suggesting such alterations occur relatively late in the pathogenesis of malignancy. Alternatively, the precursor abnormalities studied could represent genetically distinct lesions that had not given rise to the corresponding invasive carcinoma present in the resected specimen. However, the number of cases with precursor squamous lesions was too low in our study to draw a meaningful conclusion. Interestingly, reduced protein expression was relatively uncommon in lymph node metastasis, suggesting these alterations are unlikely to play an important role in the development of metastatic potential; however, the sample numbers with regional node spread were small and a larger study would be required to validate this finding.

Although reduced expression of DNA repair proteins has been demonstrated in a variable proportion of NSCLCs, the prognostic significance of these alterations has only very rarely been reported. In our study, we have been unable to demonstrate any association between alterations in expression of the DNA repair proteins MLH1, MSH2 and MGMT and survival. Similarly, in a study of non-smoking Taiwanese female patients with NSCLC, neither MLH1 expression nor promoter hypermethylation were significantly associated with prognosis.^13^ Although the authors were able to demonstrate an association between MSH2 promoter methylation and shorter overall survival, results of the relationship between MSH2 protein expression and survival were not reported.^13^ Other studies examining the methylation status of MLH1 and MGMT gene promoters have not shown any correlation with overall survival.^14^ This is in contrast to colorectal carcinomas, where defective MMR protein function is a beneficial prognostic feature.^1^,^26^,^27^

## Abbreviations

ADC: adenocarcinoma
BAC: bronchioloalveolar carcinoma
CI: confidence interval
HR: hazard ratio
LCC: large cell carcinoma
MGMT: methyl guanine DNA methyltransferase
MMR: mismatch repair
NSCLC: non-small-cell lung cancer
SCC: squamous cell carcinoma
TMA: tissue microarray
